# Actinomycosis of the nasal cavity

**DOI:** 10.1016/j.bjorl.2021.05.003

**Published:** 2021-05-25

**Authors:** Kyung Seok Park, Dong Hoon Lee, Sang Chul Lim

**Affiliations:** Chonnam National University Medical School & Hwasun Hospital, Department of Otolaryngology-Head and Neck Surgery, Hwasun, South Korea

**Keywords:** Nasal cavity, Actinomycosis, Antibiotics, Endoscopic surgery, Maxillary sinus

## Abstract

**Introduction:**

Actinomycosis of the nasal cavity is very rare.

**Objective:**

The purpose of this study was to investigate the clinical features, treatment methods, and treatment results of actinomycosis of the nasal cavity in our hospital.

**Methods:**

We retrospectively enrolled 11 patients with histopathologically identified actinomycosis of the nasal cavity from January 2010 to May 2020.

**Results:**

This study included five males and six females. The most common symptom was purulent nasal discharge (36.4%). Nasal actinomycosis occurred in the maxillary sinus in 5 (45.5%) patients, the ethmoid sinus in two, the hard palate in two, the frontal sinus in one, and the nasal septum in one. After surgery, intravenous administration of antibiotics was performed on average for 7.4 days and oral antibiotics were prescribed for about 120.5 days. The clinical characteristics of the patients with nasal actinomycosis and the duration of antibiotic usage were not significantly different. Trauma was significantly associated with repeated nasal actinomycosis infections (*p* < 0.05).

**Conclusion:**

Actinomycosis of the nasal cavity should be suspected when a patient with chronic sinusitis does not respond to medical therapy and has a history of dental treatment, local surgery or radiation therapy. Nasal can be sufficiently treated with antibiotics and endoscopic surgery.

## Highlights

• Actinomycosis of the nasal cavity is very rare.
• Actinomycosis of the nasal cavity should be suspected when a patient with chronic sinusitis does not respond to medical therapy.
• Nasal actinomycosis can be sufficiently treated with antibiotics and endoscopic surgery.

## Introduction

Actinomycosis is a rare human disease usually caused by *Actinomyces israelii*.^1^, ^2^, ^3^, ^4^ Actinomycosis is classified into cervicofacial, thoracic, abdominopelvic, and central nervous system involvement.^1^, ^2^, ^3^, ^4^ Although more than a half of all reported cases of actinomycosis involve the cervicofacial region, nasal cavity involvement is extremely rare, and only a few cases have been reported.^1^, ^2^, ^3^, ^4^, ^5^, ^6^, ^7^, ^8^, ^9^, ^10^ Therefore, the clinical characteristics and treatment protocols for nasal actinomycosis have not been established. The purpose of this study was to investigate the clinical features, treatment methods, and treatment results of actinomycosis of the nasal cavity in our hospital.

## Methods

This study was conducted following approval from the Institutional Review Board of CNUHH (CNUHH-2020-141). We retrospectively enrolled 11 patients with histopathological diagnosis of actinomycosis of the nasal cavity from January 2010 to May 2020. The patients’ clinical data were reviewed for age, sex, underlying diseases, dental treatment, trauma, repeated infections, symptoms, duration of symptoms, location, preoperative biopsy, paranasal sinus computed tomography (PNS CT) findings, preoperative diagnosis, surgical method, microbiological culture results, postoperative treatment, complications, and recurrence.

All patients underwent PNS CT before surgery to assess the extent of the lesions and treatment planning. The PNS CT findings were analyzed for opacity, calcification, and bone destruction. The opacity of the PNS CT findings was divided into partial and total opacity. All patients underwent endoscopic surgery, and the histopathological examinations confirmed the diagnosis of actinomycosis.

## Results

The clinical data from 11 patients with nasal actinomycosis are summarized in Table 1. This study included five males (45.5%) and six females (54.5%). The patient ages ranged from 36 to 83 years, with an average of 63.9 ± 14.5 years. Four patients had underlying systemic arterial hypertension, four had diabetes mellitus, two had nasal malignancies (NK/T-cell lymphoma and maxillary transitional carcinoma), and one had liver cirrhosis. Two patients had history of dental treatment on the lesion region. Three patients underwent surgery (brain aneurysm [n = 1] and endoscopic sinus surgery [n = 2]) on the lesion site, and bone destruction was found on PNS CT. Two patients with nasal malignancies were treated with 4000 cGy (NK/T-cell lymphoma) and 6480 cGy (maxillary transitional carcinoma) Radiation Therapy (RT). Three patients had repeated infections in the same area.

**Table 1:** Clinical data of eleven patients with actinomycosis of the nasal cavity.

Age (Yr/sex)	Underlying disease	Dental treat	Trauma	Repeated infection	Symptom (duration, Mo)	Site	Location	Opacity	Bone dest	Preop. Ds.	IV Anti (duration, D)	Oral Anti (duration, D)	F/U	Recur
65/F	HTN, DM				PND (1)	R	Maxillary	T		Fungus sinusitis	Penicillin (3)	Penicillin (14)	121	
59/F					PND (0.5)	L	Ethmoid	P		Chronic sinusitis	Penicillin (10)	Penicillin (51)	96	
36/M					Anosmia (4)	L	Septum	T		Mucocele	Cepha (4)	Penicillin (126)	22	
60/M	DM				Headache (1)	L	Ethmoid	P		Fungus sinusitis	Cepha (3)	Penicillin (35)	5	
82/F			Surgery	O	Headache (1)	R	Frontal	P		Fungus sinusitis	Cepha (4)	Penicillin (41)	16	
66/M	Lymphoma		RT	O	Foul odor (10)	B	Hard palate	P	O	Actinomycosis	Cepha (11)	Penicillin (221)	53	
54/M	Maxillary ca		Surgery,RT	O	Bleeding (0.5)	R	Maxillary	P	O	Actinomycosis	Penicillin (8)	Penicillin (397)	83	O
83/F	HTN, DM	O			PND (5)	L	Maxillary	T	O	ONF	Cepha (18)	Penicillin (42)	27	
83/F	HTN, DM				Pain (4)	L	Hard palate	T	O	Actinomycosis	Cepha (15)	Penicillin (191)	25	
53/F		O			PND (3)	R	Maxillary	P		Fungus sinusitis	Cepha (2)	Cepha (50)	14	
62/M	HTN, LC		Surgery		Pain (2)	L	Maxillary	T		Mucocele	Cepha (3)	Penicillin (157)	13	

Yr, years; Mo, months; Dest, destruction; Preop. Ds, preoperative diagnosis; IV Anti, intravenous antibiotics; D, days; Oral Anti, oral antibiotics; F/U, follow-up; F, female; M, male; HTN, hypertension; DM, diabetes mellitus; Ca, carcinoma; LC, liver cirrhosis; RT, radiation therapy; PND, purulent nasal discharge; R, right; L, left; B, both; T, total opacity; P, partial opacity; ONF, oroantral fistula; Cepha, cephalosporin.

**Figure 1:**
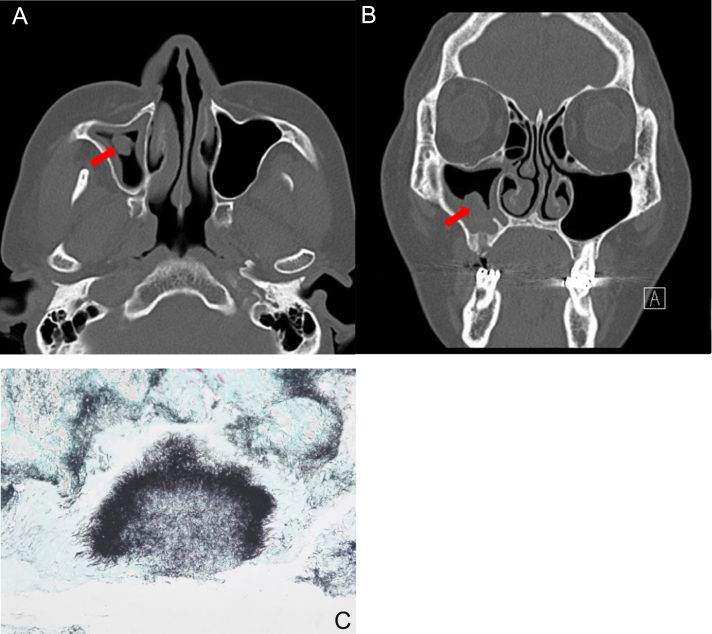
A 53-year-old female patient with actinomycosis of the right maxillary sinus. Axial (A) and coronal, (B) CT scans show partial opacity and fungal ball-like material (red arrow) in the right maxillary sinus. (C) Histopathologic findings show characteristic sulfur granules with chronic inflammation (methenamine silver stain, ×100).

The most common symptoms were purulent nasal discharge (n = 4, 36.4%), followed by headache (n = 2), pain (n = 2), anosmia (n = 1), foul odor (n = 1), and nasal bleeding (n = 1). The duration of symptoms was 2.9 ± 2.8 months (range, 0.5–10 months). Nasal actinomycosis occurred in the maxillary sinus in 5 (45.5%, Fig. 1) patients, in the ethmoid sinus in two patients, the hard palate in two patients, in frontal sinus in one patient, and the nasal septum in one patient. Among the eleven lesions, 4 (36.4%) were in the right nasal cavity and 6 (54.5%) were in the left side. One case (9.1%) of hard palate actinomycosis occurred in both sides.

In the PNS CT, six patients showed partial opacity and five patients showed total opacity. No patients had calcification within their lesions. Bone destruction due to the disease was observed in four patients, except for two patients who had previously experienced surgical bone destruction. A preoperative biopsy was performed in two patients and diagnosed as actinomycosis and granulation, respectively. The preoperative diagnosis was considered to be fungal sinusitis in four patients, actinomycosis in three, mucocele in two, oroantral fistula in one, and chronic sinusitis in one patient.

All patients underwent endoscopic surgery. Microbiological culture testing was performed in five patients, but none of them were cultured with *Actinomyces*. After surgery, mainly cephalosporin was administered intravenously for an average of 7.4 ± 5.5 days (range, 3–18 days). Subsequently, oral penicillin was prescribed for an average of 120.5 ± 115.7 days (range, 14–397 days). The duration or dose of antibiotic treatment was determined by comprehensively checking the severity of the disease, the patient’s symptoms, and the results of endoscopy and imaging tests, and finally consulting with the Department of Infectious Medicine.

The mean follow-up period after surgery was 43.2 ± 39.4 months, with a range of 5–121 months. One patient relapsed four years after the first operation and improved through reoperation and antibiotic treatment. Since then, there have been no cases of relapse.

## Discussion

First, we tried to determine the relationship between the clinical characteristics of nasal actinomycosis and the duration of antibiotic use. We analyzed the statistical significance of age, sex, underlying diseases, dental treatment, trauma, repeated infections, symptoms, duration of symptoms, location, PNS CT findings, and the duration of antibiotic use. However, the clinical characteristics of nasal actinomycosis and the duration of antibiotic therapy showed no statistically significant differences (*p* > 0.05).

Second, we investigated the relationship between the clinical characteristics of nasal actinomycosis and repeated infections. Only trauma, such as surgery or RT, was significantly associated with repeated infections of nasal actinomycosis (*p* < 0.05). Therefore, it is necessary to consider the possibility of nasal actinomycosis if a repeated infection occurs in a previous surgical or RT area.

The clinical symptoms of nasal actinomycosis were consistent with chronic sinusitis by other organisms such as bacteria or fungus.^1^, ^4^, ^7^ In this study, it was difficult to diagnose actinomycosis before surgery because the chief complaint was nonspecific nasal symptoms such as purulent nasal discharge. In the three patients who were thought to have actinomycosis before surgery, actinomycosis was considered first because a necrotic portion and bone destruction were observed in the physical examination and PNS CT. In addition, they complained of unique symptoms, such as foul odor or nasal bleeding.

Actinomycosis of the nasal cavity occurs mainly in the maxillary sinus.^1^, ^5^ Studies reported that PNS CT images of nasal actinomycosis found showed opacities in the paranasal sinus, focal calcified lesions, or focal areas of bone destruction.^1^, ^2^, ^3^, ^4^, ^5^, ^6^, ^7^, ^8^, ^9^ However, there are no uniform characteristics that can absolutely determine the diagnosis of nasal actinomycosis on radiologic examinations.^6^ A definitive diagnosis of actinomycosis of the nasal cavity is made by microbiologic or histopathologic findings.^1^, ^2^, ^3^, ^4^, ^5^, ^6^, ^7^, ^8^, ^9^, ^10^ However, culturing *Actinomyces* is difficult due to the anaerobic nature of the organism.^2^, ^3^, ^4^, ^6^, ^7^, ^8^ Microbiological culture testing was performed in five patients, but none of them cultured *Actinomyces*. Sulfur granules on removed specimens can help diagnose actinomycosis.^2^, ^3^, ^4^, ^7^, ^8^ However, the diagnostic rate of sulfur granules is about 30% and can be found in other diseases such as nocardiosis.^3^, ^4^, ^6^ Therefore, the diagnosis of actinomycosis of the nasal cavity should be based on clinical examination, microbiologic or histopathologic findings, and sulfur granules.^4^

A combination of medical and surgical treatment is recommended for nasal actinomycosis.^1^, ^2^, ^3^, ^4^, ^5^, ^6^, ^7^, ^8^, ^9^, ^10^ Actinomycosis grows best in an anaerobic environment, so the surgical removal of the involved tissues and endoscopic establishment of a patent natural sinus ostium may be essential for the successful treatment of nasal actinomycosis.^1^, ^4^, ^9^, ^10^ The standard antibiotic remains penicillin, but doxycycline, erythromycin, and cephalosporin may be used.^1^, ^2^, ^3^, ^4^, ^5^, ^6^, ^7^, ^8^, ^9^, ^10^ We used penicillin and cephalosporin in consultation with the Department of Infectious Medicine. However, the recommended treatment regimens vary from two weeks to several months.^1^, ^4^, ^7^, ^9^^,^^10^ Recently, the duration or dosage of antibiotic therapy has been determined according to the severity of the disease and the response to treatment.^4^, ^5^, ^7^, ^9^^,^^10^ Many studies have reported that short-term antibiotic treatment is sufficient for treating non-invasive actinomycosis of the nasal cavity.^7^, ^9^, ^10^

The prognosis for nasal actinomycosis is very good,^1^, ^2^, ^3^, ^4^, ^5^, ^6^, ^7^, ^8^, ^9^, ^10^ and in this study, the symptoms improved in all patients. However, long-term follow-up is necessary because the infection can recur even after several years.

## Conclusion

Actinomycosis of the nasal cavity is very rare and difficult to distinguish from chronic sinusitis caused by other microorganisms based on clinical features or radiologic examinations. Actinomycosis of the nasal cavity should be suspected when a patient with chronic sinusitis does not respond to medical therapy and has a history of dental treatment, surgery, or RT. Nasal actinomycosis is sufficiently treated with antibiotics and endoscopic surgery.

## Conflicts of interest

The authors declare no conflicts of interest.
